# Optimizing telemedical care in neurological outpatients by characterizing the patients’ needs in the physician–patient relationship—content analysis of guideline-based interviews

**DOI:** 10.1186/s12883-021-02329-y

**Published:** 2021-07-24

**Authors:** Till Hamann, Stella Lemke, Peter Kropp, Florian Rimmele, Tim P. Jürgens, Fabian Frielitz

**Affiliations:** 1 Department of Neurology, Headache Center North-East, University Medical Center Rostock, Rostock, Germany; 2 Department of Institute for Social Medicine and Epidemiology, University Medical Center Lübeck, University of Lübeck, Lübeck, Germany; 3 Department of “Medical Psychology and Sociology”, Headache Center North-East, University Medical Center Rostock, Rostock, Germany; 4 Department of “Center for Population Medicine and Health Services Research (ZBV)”, University Medical Center Lübeck, Lübeck, Germany

**Keywords:** Neurological patients, Recall-Service, Telemedical care, Evaluation portals

## Abstract

**Background:**

The use of new concepts in patient care, such as video-consultations, reminder systems, and online evaluation portals, is becoming increasingly important in the physician–patient relationship and outpatient care. This study examines the acceptance of these approaches in a neurological setting and determines the patients’ preferences.

**Methods:**

We analyzed 16 guideline-based qualitative interviews with neurological patients using qualitative content analysis (inductive category formation).

**Results:**

The patients commented on the benefits and challenges of integrating new concepts of medical care. They identified advantages of telemedical care, including time savings (7 of 16; 43,8%) for both the patient and the physician, the prospect of more intensive (4 of 16; 25%) care, and the possibility for a quick response in case of urgent needs (3 of 16; 18.8%). Several challenges were reported, such as the limitations for patients with psychiatric (2 of 16; 12.5%) or complex diseases (4 of 16; 25%) and limited options for diagnostic procedures (such as physical examination (4 of 16; 25%)).

For individual neurological patients' needs, telemedical and telecommunication structures could be discussed, which support the patients' specific requirements, such as answering questions while having a recall (2 of 16; 12.5%) and avoiding the journey (8 of 16; 50%). Also, patients are rejecting evaluation portals and are skeptical of telecare in the treatment of neurological diseases.

**Discussion:**

The perception of telemedical care and the successful integration of new medical care concepts depend on fulfilling the individual patient’s needs. Regardless of the preferred nature of physician–patient interactions, there are specific instruments that can intensify the relationship. These individual needs of the patients must be inquired about and accommodated for.

**Conclusions:**

For the first time, we could characterize the properties of optimal telemedical care for neurological patients. Interviews like the ones we conducted should be repeated during and after the pandemic, referring to our results and compare.

**Supplementary Information:**

The online version contains supplementary material available at 10.1186/s12883-021-02329-y.

## Introduction/background

In the neurological outpatient department at the University of Rostock, Germany, we treat patients with several forms of neurological disorders like headache, multiple sclerosis, and Parkinson’s disease. The tertiary academic center is located within a regional capital surrounded by a rural, sparsely populated landscape, resulting in the requirement for many patients to travel in order to seek expert medical advice. As waiting times for an appointment have grown longer over the last years, a missed appointment can negatively affect the patient’s medical condition.

Medical care in sparsely-populated areas is becoming increasingly difficult due to an ongoing rural exodus. The density of specialists in Germany has steadily decreased in recent years. In 2015, the density of neurologists, psychiatrists, and specialists in psychiatry and psychotherapy in large cities (as defined by the “Bundesamt für Bauwesen und Raumordnung”) were 1:13,745. Outside these large cities, the ratio was only 1:31,183 [1]. At the same time, the proportion of elderly increases as the cause of the demographic change [2]. The risk of neurological diseases increases with the age of the patients (e.g., the prevalence of dementia [3] or Parkinson’s disease [4]). Limited facilities and increasing need for neurological care in an aging population with decreasing mobility in rural regions could imply a greater need for the extensive use of telemedical approaches such as expert chats, video, or telephone consultations, as has been shown before [5–7].

Because innovations in communication technology and clinical data systems are emerging rapidly, there is enormous opportunity. At the same time, substantial uncertainty persists about the consequences of these tools and platforms for the patient care and provider experiences [8, 9].

While socioeconomic considerations (such as reimbursement) and medicolegal aspects (such as the legal status of telemedical examinations in regularly scheduled outpatients) have prevented the widespread use of telemedicine in everyday practice previously, the COVID-19 pandemic caused the need for minimizing physical doctor-patient contact [10]. As a consequence, the implementation of telemedical applications has been accelerated [11]. However, the use of telemedical services in everyday clinical practice still varies significantly between countries due to the different technological infrastructures and health care systems [12]. Therefore, this pandemic, despite all its negative medical and social implications, also represents an opportunity for telemedicine [13, 14].

The recent COVID19 pandemic has quickly increased the necessity to adopt digital tools and integrate new concepts in medicine, especially in the outpatient sector, in order to reduce physical contact [15]. To optimize the use of a new armamentarium of digital tools affecting the doctor-patient relationship, we aimed to characterize the individual patient’s needs in a neurological setting. We designed three central research questions:

- How do the patients evaluate reminder systems and the contact via phone call and text message?
- What are the requirements, chances, and risks of telemedical treatment?
- How do the patients assess the use of online evaluation-portals of physicians?

## Material and methods

**Fig. 1:**
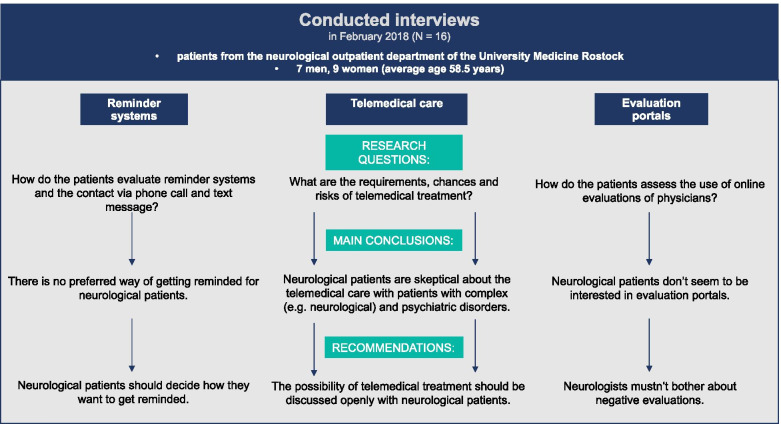
Design and Research Questions

To answering the research questions (Fig. 1), we used a qualitative approach and conducted interviews based on standard interview guidelines [16]. The patients were recruited from the Neurological Outpatient Department for movement disorders and pain in February 2018. The interviews were conducted with patients over the age of 18 years. Patients with dementia were excluded. The regional Ethics Committee of the University of Rostock approved this study (No. A 2017–0186).

Before the interview began, patients had to sign an informed consent form, and they were briefed about the objectives of the study and possible ethical concerns. The patients were asked to share their thoughts, including expectations and concerns regarding the use of reminder systems, online evaluation portals, and the use of telemedicine.

Based on our research questions, the following questions were asked in the guideline-based interviews. The full coding guide, which has been developed for this study, is shown in “supplementary files” as “Additional file 1”.

1. How do you rate the reminder systems? What are the main advantages of a phone call and a text message?
2. What do you think of online evaluation portals about doctors? What are the advantages and disadvantages?
3. Where do you see opportunities and risks in telemedical care? Are there requirements that have to be met for telemedical treatment?

The interviews were conducted in German, written down verbatim as per Kuckartz's transcription of content and semantics [17], and analyzed and evaluated with the help of the program "QCAmap" [18]. The results were translated into English.

Based on the data and objectives of the study, a qualitative content analysis according to Mayring’s [19] was chosen to answer the research questions with the help of the technique of inductive category formation. Subsequently, the categories were formed along the interview material using specific selection criteria.

For example, for answering the first research question, all text passages in which the patient in any way referred to reminder systems were categorized (selection criterion). The categories were formed on the level of "Concrete positive/negative aspects of the reminder systems" (level of abstraction). This procedure was applied analogously for the other central questions. Multiple answers were counted.

The Qualitative Content Analysis resulted in a category system with its respective frequencies. The interviews were also analyzed by a second researcher (intercoder) after the first coder, using the same technique Because of an 85% compliance, we decided to use the results of the first coder, who also conducted the interviews.

## Results

### Interviews

The 16 interviews were conducted with patients of our neurological outpatient clinic. The duration of each interview was between 7 and 17 min. A total of seven men and nine women were interviewed. The average age of the subjects was 54.9 years (median: 58.5 years, range: 27 to 87 years, standard deviation: 19.4 years).

### Reminder systems

We collected different advantages and disadvantages of reminder systems. Twenty text passages were coded to positive aspects; hindering factors were cited in 28 text passages (Table 1). The benefits can be summarized into categories (such as avoidable causes for missing the appointment, advantages for the patients). “Avoidable causes of missing the appointment” was the most frequently collected category. The most important avoidable cause, which can be prevented, was missing an appointment by forgetting (8 of 16 interviews; 50%). To structure everyday life was also mentioned as a leading positive advantage of the reminder systems (3 of 16 interviews; 18.8%). Nevertheless, for almost 50% of the neurological patients, no added value of the reminder systems could not be directly recognized because they note down the appointments independently or are not forgetful (ever 7 of 16 interviews; 43.8%). Aside from the category (“Not necessary”), also another one (“Not practical”) could have been collected frequently.

**Table 1:** Inductive category formation regarding the advantages and disadvantages of a reminder service

Category		Absolute mentions in the interviews; *N* = 16	Relative mentions in the interviews in %
**Benefits**			
Avoidable causes of missing the appointment			
**B2**	The patient forgets the appointment	8	50
**B1**	The patient doesn’t make a note of the appointment	1	6.3
Advantages for the patient			
**B6**	A reminder improves planning for everyday life	3	18.8
**B7**	A reminder means that the patient does not have to pay attention to the appointment himself	2	12.5
**B4**	After a new appointment has been made, the waiting times are very long	1	6.3
**B8**	For the patient, the appointment can be very important	1	6.3
Unavoidable causes of missing the appointment			
**B3**	The patient can’t keep the appointment due to an illness	1	6.3
Advantages for the physician’s office			
**B5**	Reduction of waiting times for the physician’s office, because the patients will miss the appointments less	1	6.3
**Challenges**			
Not necessary			
**C1**	The patient says that he/she isn’t forgetful	7	43.8
**C4**	The patient does make a note of the appointment	7	43.8
**C2**	The patient uses his/her smartphone for reminding	1	6.3
**C5**	The patient only forgets appointments which aren’t important	1	6.3
Not practical			
**C6**	The patient says that it will annoy him/her	2	12.5
**C7**	The patient is very busy	2	12.5
**C8**	The patient doesn’t check his phone as much as necessary	2	12.5
**C3**	It hasn’t worked in the past	1	6.3
Other reasons			
**C9**	The patient is afraid of crime	1	6.3

In exploring different forms of reminder systems, advantages of a text message (e.g., SMS or e-mail) and a telephone call were named. Altogether 13 advantages were mentioned for text messages and 10 advantages for the reminder call (Table 2). For the calls, the personal form of contact was emphasized (3 of 16 interviews; 18.8%), or the fact that even a missed phone call will not be forgotten (4 of 16 interviews; 25%), the main advantage of the text message was the possibility to read it at a convenient time for the patient (4 of 16 interviews; 25%) and that it will be read in any case (4 of 16 interviews; 25%). One patient described this as follows:

*“I can call up an e-mail when I want to […], and I have to react immediately to such a call.”*

**Table 2:** Inductive category formation regarding the advantages of a phone call and a text message

Category		Absolute mentions in the interviews, *N* = 16	Relative mentions in the interviews in %
Advantages of a phone call			
**D3**	The patients don’t forget a phone call or a missed phone call	4	25
**D4**	A phone call is more personal	3	18.8
**D6**	The patient may ask several questions during a phone call	2	12.5
**D1**	A phone call is faster than a text message	1	6.3
Advantages of a text message			
**E1**	A text message will be read in any case	4	25
**E4**	The patient decides when he/she reads the text message	4	25
**E2**	A text message doesn’t bother the patient	1	6.3
**E3**	A text message is faster than a phone call	2	12.5
**E5**	The patient knows for sure that the reminder comes from the physician	1	6.3

In conclusion, partially very similar arguments for both types of reminder could have been collected. For example, the argument, that the one way of the reminding would be faster than the other, could have been collected for the phone call and the text message (D4 and E3). Also, both of them won’t be forgotten (D3 and E1). A "better way of reminding" was not mentioned.

### Telemedical care

The patients mentioned the advantages and disadvantages of telemedical care. Especially in the course of the demographic change (e.g., for now sparsely-populated landscapes, such as Mecklenburg-Pomerania [20]), the patients emphasized the strengths of telemedicine.

Thirty-seven aspects could be determined, which were seen as advantages of telemedical treatment (Table 3). Time-saving for patients (7 of 16 interviews; 43.8%) and avoiding the journey (8 of 16 interviews, 50%) were found most often. Once the participants also mentioned time-saving for physicians. Twenty-one of these positive aspects were mentioned, 8 of which by the younger patients (< 58 years), 16 by the older (*p* = 0,5).

**Table 3:** Inductive category formation regarding the advantages and disadvantages of telemedical treatment

Category		Absolute mentions in the interviews, *N* = 16	Relative mentions in the interviews in %
**Benefits**			
Advantages for the patient			
**L2**	Telemedicine saves time	7	43.8
**L1**	Telemedicine is practical for long journeys and patients with low mobility	8	50
**L4**	Telemedicine offers intensive and better care	4	25
**L8**	Telemedicine enables quick action in emergencies	3	18.8
**L5**	Telemedicine is compatible with work	3	18.8
**L7**	Telemedicine is practical for chronic illnesses	2	12.5
**L6**	Telemedicine offers treatment options that can be varied over time	1	6.3
**L9**	Telemedicine is practical for getting a second opinion	1	6.3
**L11**	Telemedicine is practical for avoiding full waiting rooms	1	6.3
Positive aspects for the physician–patient relationship			
**L10**	Telemedicine is practical for improving the trust between physician and patient	1	6.3
Advantages for the physician			
**L3**	Telemedicine saves time	1	6.3
**Situations where the use of telemedicine makes sense**			
**N3**	Discussion of results	8	50
**N1**	Routine checks for chronic diseases	3	18.8
**N5**	Planning therapy	2	12.5
**N4**	Anamnese	1	6.3
**Challenges**			
Reasons why patients are skeptical about telemedicine			
**M7**	Telemedicine is too impersonal	7	43.8
**M4**	The patients feel better with doctors they know	3	18.8
**M9**	Telemedicine depends on technical requirements	2	12.5
**M10**	The patients are afraid of deception	2	12.5
**M14**	Telemedicine can’t inspire trust between doctor and patient	1	6.3
**M12**	Telemedicine uses too many technical vocabulary	1	6.3
Reasons why telemedicine isn’t helpful in general			
**M5**	Telemedicine doesn’t offer optimal care of the patient	6	37.5
**M16**	While using telemedicine there’s no possibility for physical examination or invasive diagnostics	4	25
**M8**	In personal contact the patient can focus better on his/her illness	2	12.5
**M13**	Telemedicine can lead to misunderstandings between physician and patient	1	6.3
Illnesses and situations in which telemedicine isn’t helpful			
**M2**	Telemedicine isn’t helpful for finding the correct diagnosis	6	37.5
**M3**	Telemedicine isn’t helpful for complex illnesses	4	25
**M6**	Telemedicine isn’t helpful for psychiatric illnesses	2	12.5
**M11**	Telemedicine isn’t helpful for acute illnesses	1	6.3
**M15**	Telemedicine isn’t helpful for palliative illnesses	1	6.3

Other possible challenges of telemedical counseling (Table 3), besides the impression of impersonal counseling (7 of 16 interviews; 43.8%), or the lack of possibilities of physical examination were mentioned (4 of 16 interviews; 25%). One patient described this as follows:

*“[...] if I’m ill and I go online with the doctor, he cannot examine me and, e.g., look down my throat, [...] Then I can also ask “Dr. Google” in principle. Diagnosing is more difficult for the physician.”*

Furthermore, other hindering factors could be categorized (Table 3). Besides these "general" problems of telemedicine, the neurological patients also reported concrete situations where they would prefer regular contact. Determining the correct diagnosis, psychiatric diseases, emergencies, and "complex" diseases were often mentioned here.

However, patients also described situations, in which telemedical care would be indicated. Most frequently, the "discussion of results" was named (8 of 16 Interviews; 50%), but also emergencies (3 of 16 Interviews; 18.8%) and routine checks for chronic diseases (3 of 16 Interviews; 18.8%) were named. Again, very similar situations are described here for entirely different questions.

### Evaluation portals

Positive (12 text passages; Table 4) and challenging (37 text passages; Table 4) aspects have been collected about online evaluations of physicians.

**Table 4:** Inductive category formation regarding the advantages and disadvantages of rating portals

Category		Absolute mentions in the interviews, *N* = 16	Relative mentions in the interviews in %
Aspects why the existing ratings aren’t helpful			
**G3**	The rating portals aren’t reliable	8	50
**G1**	The rating portals don’t show a unified opinion	7	43.8
**G5**	The rating portals don’t show trustworthy comments	3	18.8
**G6**	The patient is also responsible for the success of the treatment	1	6.3
Aspects why ratings aren’t helpful in general			
**G2**	The personal impression is more important than reviews	7	43.8
**G4**	Personal recommendations are more important than reviews	3	18.8
**G8**	The patient doesn’t want to look for a new physician	3	18.8
Aspects making existing ratings more helpful			
**H3**	Reasoned ratings provide an impression of the physician	4	25
**H2**	Many similar ratings provide an impression of the doctor	2	12.5
Aspects about which ratings make a statement			
**H1**	Ratings give a first impression of an unknown physician	3	18.8
**H4**	Ratings give an overview of the physician’s quality	1	6.3
**H5**	Ratings give an overview about many aspects	1	6.3

All in all, we collected aspects of why evaluations are not helpful in general and why the existing evaluations are not helpful. The lack of reliability of the portals is named in 50% of the interviews. A participant states:

*“Because also some people write like they are on top of the world, or in the depths of despair.”*

Furthermore, the personal impression is valued higher than an evaluation (7 of 16 interviews; 43.8%):

*“You have to go to the doctor yourself, have the first talk, and then see how it works.”*

Only 12 text passages said something about the benefits of evaluation portals. In 4 of 16 interviews (25%), it was noted that from the patients’ point of view, a reasoned evaluation in the portals could say something about whether a doctor is a “good” or “bad” one. This argument was the most frequently mentioned one. Others like sharing their first impression of new physicians or many similar ratings provide an impression of the physicians counted less.

## Discussion

The evaluation of guideline-based interviews revealed complex answers to the research questions and the benefits and challenges of telemedical structures in the physician–patient relationship for neurological patients.

In general, the answers point to two different types of neurological patients. On the one hand, a group of patients with a pronounced need for a close physician–patient relationship, on the other hand, a group of patients who prefer a certain distance between themselves and the physician. With their different elements, telemedicine and telecommunications offer instruments for both types of patients to meet their different needs. It is essential to adjust and apply these possibilities individually to the patients' needs.

### Reminder systems

Arguments for an appointment reminder via text message as well as via call could be collected. An advantage of text messages (E3, E4) is that they are supporting the patients' desire for independence. The advantages of a call are the opposite (D4, D6), fulfilling the patients' need for a closer physician–patient relationship.

In practice, the question arises as to whether a call or a text message is more effective. Previous studies indicated that the kind of reminder system is irrelevant and that it is its presence that contributes to patient satisfaction [21], so we should stop thinking about the way of reminding. For the first time, we can confirm this for neurological patients, too. For example, the fact that an appointment will not be forgotten was categorized as an advantage for the phone call and the text message.

Reminder systems can be crucial in different ways, and effective over a great therapeutic range can be great. For example, a high vaccination rate can be achieved with the help of reminder systems [22]. Especially concerning the COVID19 pandemic, it is essential to ensure broad immunization as quickly as possible and enable everyday social and economic life.

### Telemedical care

The acceptance of telemedical care concerning more complex diseases, including chronic neurological diseases (such as multiple sclerosis or Parkinson's disease), was not always sufficient (M3). Although the interviews were conducted with patients who had been diagnosed with neurological disorders, opinions were divided. This is one of the strengths of this study, as it shows that there is no blanket approval or rejection of telemedical care for this group of patients. Patients with dementia were not included in this study. Determining the needs of this patient group can be a task for future studies.

A further problem in telemedical care for some of the study participants was the treatment of mental illnesses (M6). Reviews show that telemedical psychotherapy can also offer important advantages [23, 24]. Given the low density of medical specialists [2], such telemedical psychotherapies will be necessary because of the risk of chronisation of such diseases if professional help is not available [25]. Especially for neurological patients, these risks should be addressed more openly because patients with neurological disorders like multiple sclerosis [26] or Parkinson’s disease [27] are more frequently affected by psychiatric illnesses.

“Emergencies" were named as situations that are suitable and not suitable for telemedical care. This shows that the proper use of telemedicine depends on the patient's needs.

Independent of the primary illness, telemedical procedures were perceives as suitable for the pure discussion of findings (N3) or follow-up appointments (N1). Therefore, we should use telemedical care in such situations more often and discuss the possibilities more openly with neurological patients. For the first time, this study shows neurological patients’ openness to this form of therapy.

Almost 50% of the interviewees said that telemedical care is too impersonal (M14). This can cause the relationship to suffer. Nevertheless, former studies show no deterioration of the physician–patient relationship in patients receiving telemedical care [28, 29], a fact that has to be discussed with neurological patients in advance. This supports the thesis that neurological patients see a chance for telemedical treatment, especially for follow-up appointments.

In summary, in contrast to the reminding service, different types of patients can be identified. On the one hand, patients named advantages, which show a certain striving for independence (L9, L2). On the other hand, some categories build up a close relationship with the doctor (L10). All this increases the patients' satisfaction with the therapy, which is of decisive importance for the quality of care as well as for health benefits [30]. This study is the first to show that telemedical care can satisfy the individual needs for both types of neurological patients.

### Evaluation portals

Patients were asked about the advantages and disadvantages of evaluation portals on the internet. It turned out that for neurological patients, the online evaluations do not influence their choice of the physician. Half of the interviews showed that these portals are not taken seriously. The "lack of objectivity" is not only a feeling of the patient. In 2018, the German online portal “Jameda” was forced to delete a physician's account after a doctor took legal action against the site. The use of moderators could intervene to make these portals more trustworthy. This offers the opportunity for the doctor and the practice to improve internal quality management [31]. Support and information for the patients would help the patients get a first impression of the physician and improve the medical work to stand up to the competition. In order to increase the probability that the practice is perceived as positive, it is vital to know the types of patients described in this paper. A broad orientation of the offer improves the perception.

### Outlook

During the COVID-19 pandemic, digital care is gaining importance. The study shows that neurological patients with their various needs were aware of the opportunities and possibilities of "digital medicine". However, not only the treatment gets into focus due to the pandemic, but also the organization of the doctor's practice. Appointments get rarer, and waiting time increases. Missing such an appointment can have consequences for a patient's health. Reminder services can be helpful.

In the future, neurological patients should be encouraged to use telemedicine and telecommunications and establish the necessary infrastructure. However, this study shows that suitable telemedical tools exist for different needs of patients, which determine the choice. Central telecommunication structures can be certain typed of health advice and an individual reminder service.

## Conclusions

In summary, we could identify different needs for a physician–patient relationship in neurological patients. For both types of patients, there are telemedical instruments. In general, neurological patients seem to be sceptical about the telemedical treatment for patients with complex (e.g., neurological) and psychiatric disorders. Also, there is no preferred way of getting reminded for neurological patients. We recommend finding out to what group of patients the patient belongs to and then to adjust the telemedical care and telemedical instruments accordingly.

For the first time, the needs of neurological outpatients and conclusions for optimizing clinical pathways were identified. One of our study's strengths is that we conducted the interviews before the COVID-19 pandemic, and future studies can now take our results into account. It would be questionable to generalize our results to the present pandemic or the future. Hence, more recent interviews have to be conducted. Also, we only interviewed patients from a small urban area in northern Germany. Patient’s statements from other cities or countries might differ [14].

## Supplementary Information

**Additional file 1.** Word document, full coding guide of the interviews.

## Data Availability

The main data generated (the tables) or analyzed during this study are included in this published article. The datasets used for analyzing (the transcribed interviews) during the current study are available from the corresponding author on reasonable request. The raw interviews are password protected at www.qcamap.org Please contact TH, if you want to be a team member at qcamap. Then, you can read the whole interviews.
