# Supplementary material for: Optimizing telemedical care in neurological outpatients by characterizing the patients’ needs in the physician–patient relationship—content analysis of guideline-based interviews
Source: BMC Neurol. 2021 Jul 24;21:291. doi: 10.1186/s12883-021-02329-y (PMC8302970; doi:10.1186/s12883-021-02329-y)
Supplement: Supplementary file 1 — Additional file 1. Word document, full coding guide of the interviews. [file 12883_2021_2329_MOESM1_ESM.docx]

***Title page***

­Optimizing telemedical care in neurological outpatients by characterizing the patients’ needs in physician-patient relationship - content analysis of guideline-based interviews

Authors:

Till Hamann 1 (Corresponding author), Stella Lemke 2, Peter Kropp 3, Florian Rimmele 1, Tim P. Jürgens 1, Fabian Frielitz 4

1 Dept. of Neurology, Headache Center North-East, University Medical Center Rostock, Rostock, Germany

2 Institute for Social Medicine and Epidemiology, University of Lübeck, Lübeck, Germany,

3 Dept. of “Medical Psychology and Sociology”, Headache Center North-East, University Medical Center Rostock, Rostock, Germany

4 Dept. of “Center for Population Medicine and Health Services Research (ZBV)”, University Medical Center Lübeck, Lübeck, Germany

***Coding guide***

1. REMINDERSYSTEMS
   1. When you think about your last doctor's visits, have you ever received an appointment reminder?
   2. Yes? Was is helpful? ; No? Would that be helpful? How would you think about such a reminder? Would you use such a service?
   3. Would you give your consent to a reminder or not? Why?
   4. In what form would you prefer a reminder (e.g. phone call, SMS or e-mail)? Why?
2. INTERNET AND HOMEPAGE
   1. Do you use the internet to communicate with your doctor?
   2. Do you use the internet to inform yourself about a doctor?
   3. What influence do reviews on the Internet have on you? (e.g. "Jameda", does it make a difference whether 2 or 5 stars? Or the number of ratings given?)
   4. What aspects would you like to see on the doctor's homepage? (e.g. practice services, opening times, vacation, etc.)
   5. Would you like to make an appointment online?
   6. What do you think of the ability to order prescriptions online?
3. TELEMEDICINE
   1. Everyone talks about "Telemedicine" - what have you heard about it?
   2. Where have you come into contact with "telemedicine"? (e.g. a call from the doctor to discuss findings, or advice from the doctor via e-mail, etc.)
   3. What chances and possibilities do you think telemedicine has?
   4. In your opinion, what are the risks of telemedicine? In which cases would you rather avoid telemedicine? (e.g. with certain diagnoses / diseases, etc.)
   5. When you think about the treatment, when could telemedicine be helpful? (Think about making an appointment, treating, evaluating the success of the treatment, discussing findings / discussing medical results, etc.)
   6. What would telemedicine have to cover or what requirements would have to exist in order for you to use it? (e.g. data security, legal requirements, physical preliminary examination, etc.)
